# A direct comparison of the optically stimulated luminescent properties of BeO and Al_2_O_3_ for clinical in-vivo dosimetry

**DOI:** 10.1007/s13246-022-01155-x

**Published:** 2022-07-11

**Authors:** Benjamin Broadhead, Christopher Noble, Prabhakar Ramachandran

**Affiliations:** 1 School of Chemistry and Physics, Queensland University of Technology, Brisbane, QLD Australia; 2 Radiation Oncology, Princess Alexandra Hospital, Brisbane, QLD Australia

**Keywords:** Dosimetry, Radiotherapy, Optically stimulated luminescence, In vivo dosimetry, OSLD

## Abstract

Optically stimulated luminescence dosimetry is a relatively recent field of in-vivo dosimetry in clinical radiotherapy, developing over the last 20 years. As a pilot study, this paper presents a direct comparison between the sensitivity variance with use, stability of measurement and linearity of the current clinical standard Al_2_O_3_:C and a potential alternative, beryllium oxide. A set of ten optically stimulated luminescence dosimeters (OSLD), including five of each type, were used simultaneously and irradiated on a Versa HD linear accelerator. Having similar sensitivity, while Al_2_O_3_:C showed a relatively stable signal response from initial use, BeO was found to have a higher response to the same dose. However, BeO displayed a strong exponential decline from initial signal response following a model of $Respons{e}_{BeO}=(0.55\pm 0.05){e}^{-\left(0.40\pm 0.05\right)x}+(0.54\pm 0.01)$, reaching stability after approximately 10 irradiation cycles. BeO was shown to have potentially higher accuracy than Al_2_O_3_:C, with less variation between individual doses. Both OSLD showed good linearity between 0.2–5.0 Gy. Between these bounds, Al_2_O_3_:C demonstrated a strong linear response following the trend $Dose_{Al_{2}O_{3}, group(adj)}=(1.00\pm 0.09)x-(0.02\pm 0.04)\,{\text{Gy}}$, however beyond this showed deviation from linearity, resulting in a measured dose of $12.0\pm 0.2$ Gy at 10.0 Gy dose delivery. BeO showed strong linearity across the full examined range of 0.2–10.0 Gy with following a model of $Dos{e}_{BeO, ind}=(0.98\pm 0.01)x+(0.04\pm 0.01)$ Gy with a recorded dose at 10.0 Gy delivery as $9.9\pm 0.1$ Gy. In conclusion, BeO does show large variance in sensitivity between individual OSLD and a considerable initial variance and decline in dose–response, however after pre-conditioning and individual normalisation to offset OSLD specific sensitivity BeO provides not only a viable alternative to Al_2_O_3_:C, but potentially provide higher accuracy, precision and reproducibility for in-vivo dosimetry.

## Introduction

Optically stimulated luminescence is a property of some materials where, when exposed to ionising radiation, they ‘store’ some of this energy. When exposed to the appropriate optical light, the material is stimulated to release this stored energy as its own unique light signal. This light signal may, in turn, be used to measure the original exposed dose. A material designed explicitly for this purpose is referred to as an optically stimulated luminescent dosimeter.

There are few materials suited for optically stimulated luminescent dosimetry. For a material to meet requirements, particularly for potential clinical applications such as radiotherapy, there are several required and desirable properties such as; high radiation sensitivity, high stimulation efficiency, low effective z-number (${\text{Z}}_{\text{eff}}$), longevity of stored dose (low-fading), good reproducibility and linearity of response and importantly, simplicity and ease of use. This pilot study aims to draw a direct comparison between three of the key OSL features of beryllium oxide ceramics (BeO) and carbon-doped aluminium oxide (Al_2_O_3_:C) for clinical in-vivo dosimetry in radiation oncology, including sensitivity, stability and linearity of dose response.

One of the first materials found to have these properties and to be used for thermoluminescent (TL) dosimetry was aluminium oxide [4]. The first published paper describing Al_2_O_3_:C as an OSL material was published in 1990, where it was found that the inclusion of oxygen vacancies increased the OSL properties [1]. In 1995 Al_2_O_3_:C was compared with other crystalline materials by McKeever *et al.* and confirmed as an OSL material [6]. Currently, Al_2_O_3_:C is the most commonly used OSLD for dose measurements and as such the Landauer nanoDot™, is presently, the only commercially available OSLD designated for clinical use (Villiani *et al* [5, 11].

There may, however, be advantages to BeO ceramics for OSL dosimetry. As with Al_2_O_3_:C, BeO has both thermal and optically stimulated properties [2]. While BeO is not commonly used for clinical in-vivo dosimetry, it has been established as a radiation dosimeter in other areas [10]. Current evidence shows that BeO exhibits relatively good linearity with dose up to $\sim$ 10 Gy [3], and while beyond this, the signal begins to saturate, there is a reported deviation from linearity of less than 5% at 30 Gy with minimal long-term fading, reported as little as 1% in 6 months [10].

BeO has been shown to have a similar sensitivity to Al_2_O_3_:C, though has a greater tissue equivalency due to a low effective atomic number of ${\text{Z}}_{\text{eff}}=7.2$ [13] . However, while having similar sensitivity to that of Al_2_O_3_:C, the sensitivity and response of the ceramic have been demonstrated to vary with accumulated radiation dose and usage (read-bleach) cycles [13]. This change was reported as an increase in sensitivity for higher dose accumulation in subsequent cycles and a decrease in sensitivity over repeated cycles at lower dose. Though the decline in sensitivity for low dose was correlated to the number of cycles rather than dose accumulation, this requires further investigation [13]. An early study reports preheating BeO chips to 125 °C for 125 s to remove unstable OSL signals [3].

Another factor to be considered is the effect of a varying dose rate, as may be the case in volumetric modulated treatment deliveries. Though there are several studies analysing the direct irradiation of BeO ceramics in a research scenario, these are often from a steady or fixed activity source and there is little available information regarding the potential effects of varied fluence or dose rates as may occur in a clinical scenario. A few studies have reported that the minimum detectable dose is of the order of 10 Gy$\upmu$, however, this requires verification [13]. At the time of writing, there appears to be very little information regarding potential angular dependence on dose–response of BeO. Additionally, the effects of low energy radiation have been shown to invoke a varied response in BeO. One study using Monte-Carlo simulation and general cavity theory that there may be a significant under-response of $\sim$ 8% and $\sim$ 12% at kilovoltage x-ray energies of 50 and 100 kV, respectively, such as those that may be found in cone-beam CT (CBCT) used for patient set up [8]. In contrast, Al_2_O_3_:C has been shown to overrespond for energies below 100 kV, with one study using cavity theory reporting overresponse factor in excess of 3.5 for energies around 20 kV [7, 9].

There may also be additional dosimetry information able to be obtained in a newly discovered BeO property of thermally transferred optically stimulated luminescence. Observations have shown that heating to a temperature of $\sim$ 260 °C post bleaching results in a recovery of the OSL signal [2, 12]. This property may mean a possibility to record and quantify a ‘sum dose history’ over multiple irradiations. However, there exists a gap in the literature regarding the efficacy of this, particularly for clinical purposes where dose accuracy and precision are of great importance. The implication of this property is a possibility for recording a total patient surface dose over the course of treatment for retrospective analysis and warrants investigation.

A recently released report by the American Association of Physicists in Medicine (AAPM) titled AAPM TG 191: Clinical Use of Luminescent Dosimeters contains guidelines for assessments and corrections that may be implemented in TL and OSL dosimetry [5]. While this report focuses primarily on the most commonly used luminescent dosimeters,Al_2_O_3_:C and LiF:Mg,Ti, it does provide a base protocol to follow, including calculation of calibration coefficients and correction factors. Currently, Al_2_O_3_:C remains the most common clinically used OSLD for in-vivo dosimetry, by addressing the above gaps in research and development of a usage protocol for reading and bleach cycles may provide not only a viable OSLD alternative for in-vivo dosimetry, but also the added advantage of higher conformity to tissue equivalency and the potential for in-vivo accumulated dose measurement.

## Method

### Materials

All measurements were performed on the same set of ten OSLD, consisting of five Beryllium Oxide (Materion Thermalox® 995) ceramic chips and five carbon-doped aluminium oxide (Landauer nanoDot™). The BeO OSLD were of square geometry with $4\times 4$ mm dimensions. The Al_2_O_3_:C OSLD were circular with a 4 mm diameter. Both OSLD were approximately 0.5–1.0 mm in thickness and housed in a small, optically opaque ABS plastic housing of dimensions $10\times 10\times 2$ mm. All OSLD were used from new, and had no prior ionising radiation exposure before use.

All OSL measurements were performed using a Lexsygsmart automated TL/OSL reader (Freiberg Instruments, Germany). The system houses up to 40 OSLD at a time and is capable of reading both thermoluminescent and optically stimulated luminescent dosimeters. It utilises a closed-loop thermostatic control for heating, an internal overhead assembly containing several LED sources for stimulation at various wavelengths and photomultiplier tube for signal processing. Initial background reading before irradiation of OSLD was performed to confirm null history.

Stimulation light was provided by a 458 nm, continuous wave (CW) blue LED with an irradiance of 1 mW/cm^2^ for 1 second for reading and 100 mW/cm^2^ for a total of 300 seconds for signal bleaching. The on-board detection unit is a basic UV–VIS PMT with a sensitivity range of 280-620 nm and peak sensitivity at 420 nm. Read signal detection was taken at sampling intervals of 250 ms, providing four samples for the 1 second read window. These counts were then summed for the total count number over 1 second. Reading and bleaching of each OSLD was commenced 5–10 minutes of irradiation and occurred sequentially within the reader, taking ~360 seconds per OLSD.

Irradiations were performed on a Versa HD Elekta linear accelerator. Irradiations were performed in reference conditions using plastic water phantoms at 90 cm SSD and 10 cm depth in photon modalities. A 3D-printed ABS plastic holder, capable of positioning up to 25 indivdual OSLD plastic housings within a $5\times 5$ square arrangement was used at delivery depth to ensure alignment of the OSLD with the incident field. All OSLD when removed and replaced from housings and placed in the reader were handled using nitrile gloves, with minimal light to prevent signal loss from ambient room light. Once removed from the housing, the OSLD were placed directly in the reader, such that the average exposure time for irradiated OSLD to ambient light was less than 1 min.

### Sensitivity variance

To test sensitivity variance and potential decline from initial use, a set of ten new and unused OSLD consisting of five BeO and five Al_2_O_3_:C where exposed to repeated doses of 1 Gy delivered in a clinical setting via linear accelerator in a 6 MV photon beam. Each dose was followed by a read-bleach-read cycle of the OSL signal to determine initial irradiated OSL count and subsequent remaining background after bleaching.

### Linearity of dose–response

Using the same set of OSLD chips after the BeO had approached an observed stable sensitivity decline, all OSLD were then exposed to three repeat exposures at each of six doses: 0.2 Gy, 0.5 Gy, 1.0 Gy, 2.5 Gy, 5.0 Gy and 10.0 Gy. All OSLD were irradiated simultaneously using a 6MV clinical photon beam. OSLD were read followed by a standard bleaching process between each subsequent exposure. An average of all chips of each type at each dose was used and compared against an initial 1 Gy calibration dose to assess the linearity of response.

## Results

### Sensitivity variance

**Fig. 1:**
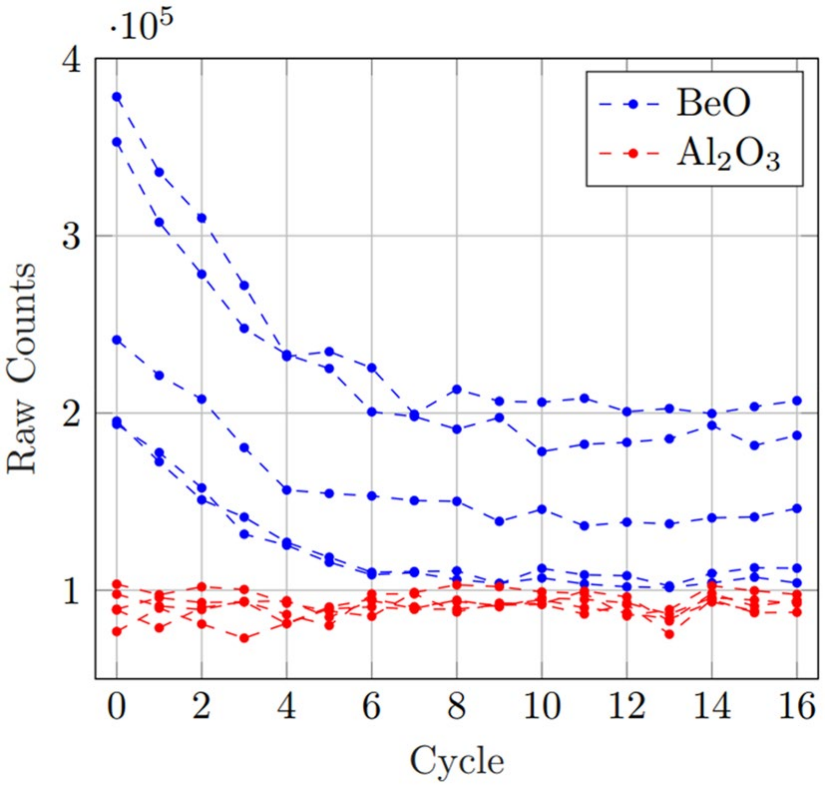
Raw counts measured from each new, unused OSLD over the series of exposure and bleach cycles. While each BeO OSLD appears to have an exponential decline from the initial response, there is a large difference between each individual sensitivity. In contrast, the Al_2_O_3_:C shows, albeit lower, a more constant sensitivity across all OSLD, with smaller individual variance

Initial response measurement for BeO OSLD showed not only a great difference between the individual sensitivities, but an apparent exponential decline in the sensitivity from initial use. This decline appeared to become much more stable after several cycles of irradiation and subsequent bleaching. In contrast to this, the Al_2_O_3_:C (nanoDot) showed only little discernible variation in gross sensitivity over all cycles. The effects of this can be observed in Figure 1.

**Fig. 2:**
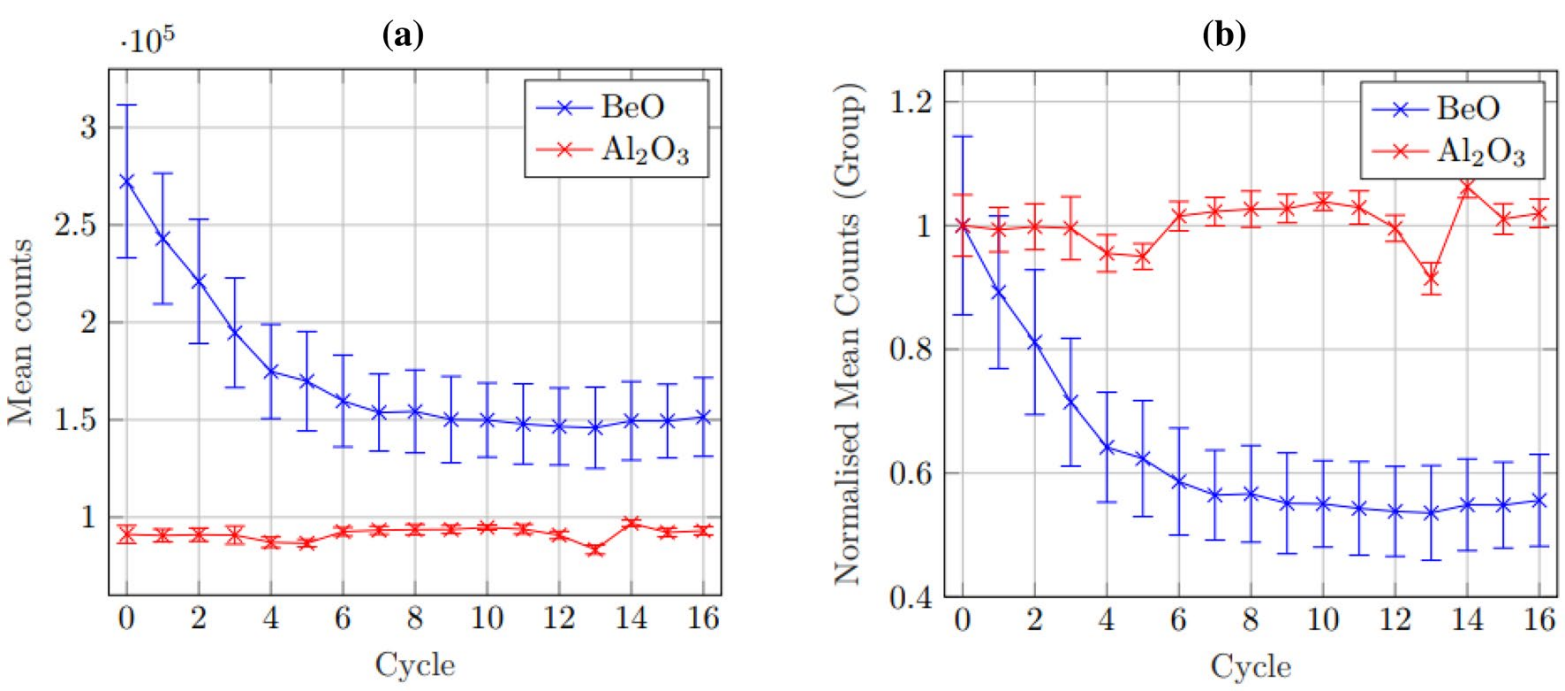
**a**) shows the average value of counts in response to 1 Gy exposure for each OSLD type. Large individual sensitivity variation observed in BeO resulted in large uncertainty in the expected value, while the Al_2_O_3_:C conform quite well. **b**) shows the same group average data, normalised to the group mean

Plotting the mean response of each OSLD type showed that while Al_2_O_3_:C remained relatively consistent in sensitivity with repeated use, BeO had a clear decline, reaching relative stability only after approximately $\ge$ 10 dose-bleach cycles. While sensitivity trends for each BeO chip followed similar paths, the individual variances resulted in large uncertainties when contrasted with the group mean. Figure 2 below shows the mean trends of both BeO and Al_2_O_3_:C when presented as an absolute average and when normalised relative to the initial group mean.

While BeO shows an exponential decline in sensitivity from the initial response and eventually approaches constancy, the variance in the gross sensitivity of any individual chip contributes to large uncertainty when dealing group averages, while the Al_2_O_3_:C remains quite consistent.

**Fig. 3:**
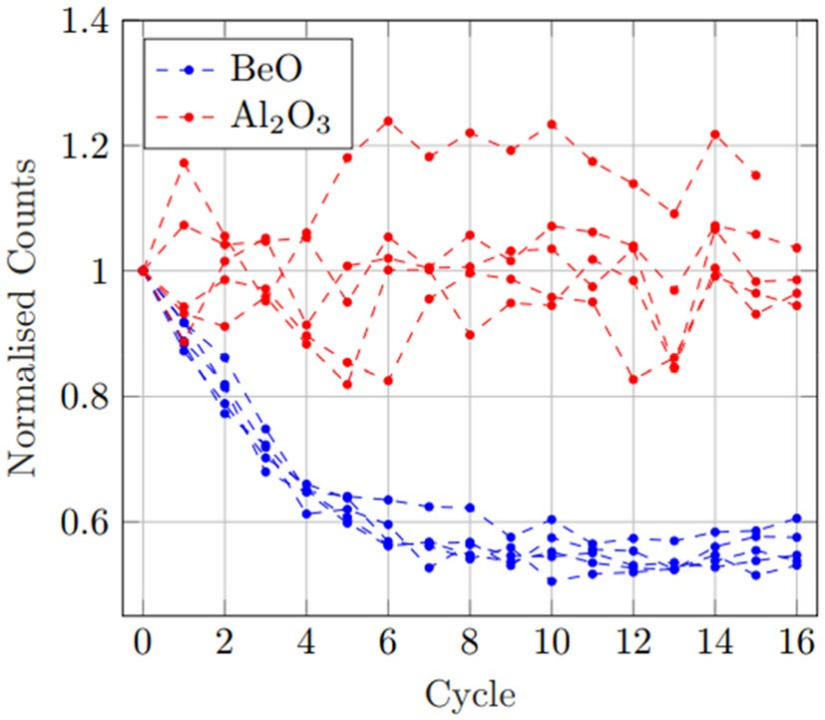
This figure shows OSLD response from new, normalised to each individual initial measurement. After OSLD specific normalisation, BeO shows much smaller variance between each OSLD with successive measurements, while Al_2_O_3_:C shows increased variance in relative response between OSLD

To offset the difference between individual BeO OSLD sensitivities, the signal was normalised to the individual OSLD initial response count. After normalisation, it appears that the BeO all follow a similar trend, with considerably smaller variance between each individual OSLD, whereas the Al_2_O_3_:C showed a very large variance in response and a much less predictable result for subsequent measurements. The individually normalised response is shown below in Figure 3.

**Fig. 4:**
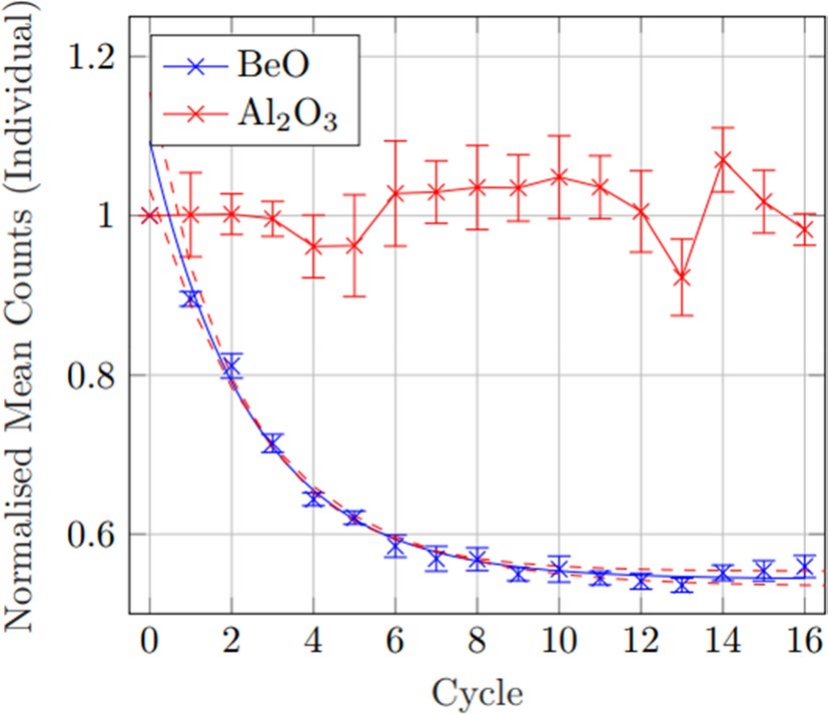
Comparison of mean OSLD sensitivity values with subsequent dose-bleach cycles when normalised to individual OSLD initial response. This method shows a large reduction in uncertainty for BeO, while an increase in uncertainty for Al_2_O_3_:C. While any model fitted to Al_2_O_3_:C would be of no discernible value due to the random nature of each individual response, a clear exponential fit can be shown for the BeO response of $Respons{e}_{BeO}=(0.55\pm 0.05){e}^{-\left(0.40\pm 0.05\right)x}+\left(0.54\pm 0.01\right)$ and ${\text{R}}^{2}=0.99$, showing very strong correlation

Taking the mean of the individually normalised counts showed much tighter uncertainty bounds for BeO, with a fitted model of $Respons{e}_{BeO}=\left(0.55\pm 0.05\right){e}^{-\left(0.40\pm 0.05\right)x}+\left(0.54\pm 0.01\right)$ and ${R}^{2}=0.99.$ Using the method of individual normalisation, Al_2_O_3_:C however showed a large increase in uncertainty, attributed to the large variance between subsequent individual measurements (Fig. 4).

### Linearity of dose response

**Fig. 5:**
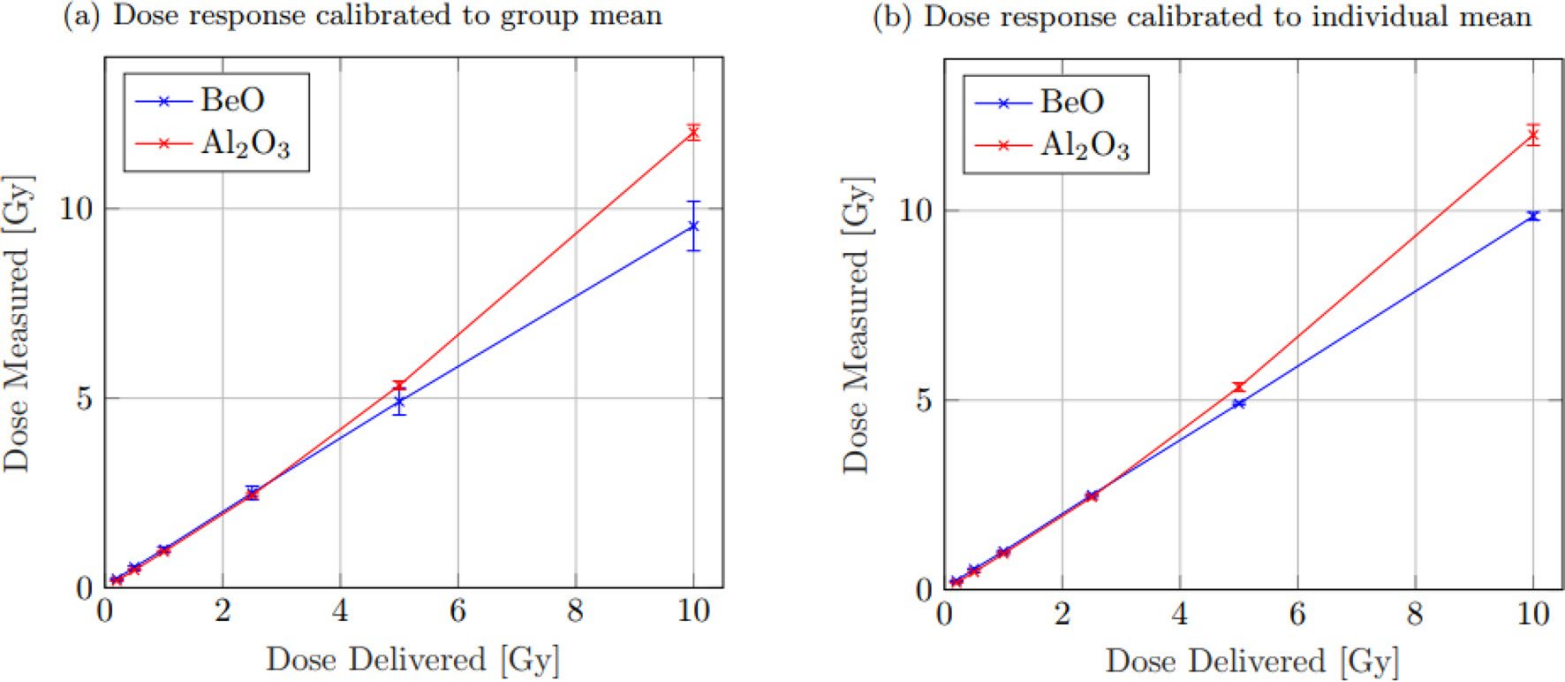
Measured dose response vs. dose delivered for both Al_2_O_3_:C and BeO. While the trend appears similar in each plot,** a**) shows the result when calibrated against the OSLD group mean, while **b**) shows the result after calibrating against the individual OSLD response

Linearity tests were performed over several dose increments ranging between $0.2-10.0$ Gy. Three irradiations were performed at each dose level, providing 15 data points for each OSLD type at each dose. The average of these 15 cumulative data points at each irradiation energy was taken and used to model and compare the linear behaviour of each within the applied range. An initial 1 Gy measurement was performed in addition to the varied dose strength to use for dose calibration. Figure 5 below shows the direct comparison of results. Figure 5a shows the trends and error bars associated with taking the mean of the total counts before individual calibration. The collected data was normalised against the mean of the initial 1 Gy calibration measurement. As with the sensitivity decline, the large variance in individual OSLD sensitivities for BeO resulted in a much higher overall variance and so greater standard error, while Al_2_O_3_:C showed a much more reproducible number. Figure 5b shows the same results, however with each OSLD instead normalised to its own individual first calibration count, prior to being averaged. In this case again, the initial normalisation to offset the individual variance in the BeO resulted in much tighter uncertainty bounds. On the contrary, the larger deviations in individual variance among Al_2_O_3_:C resulted in an increased standard error using this method.

**Fig. 6:**
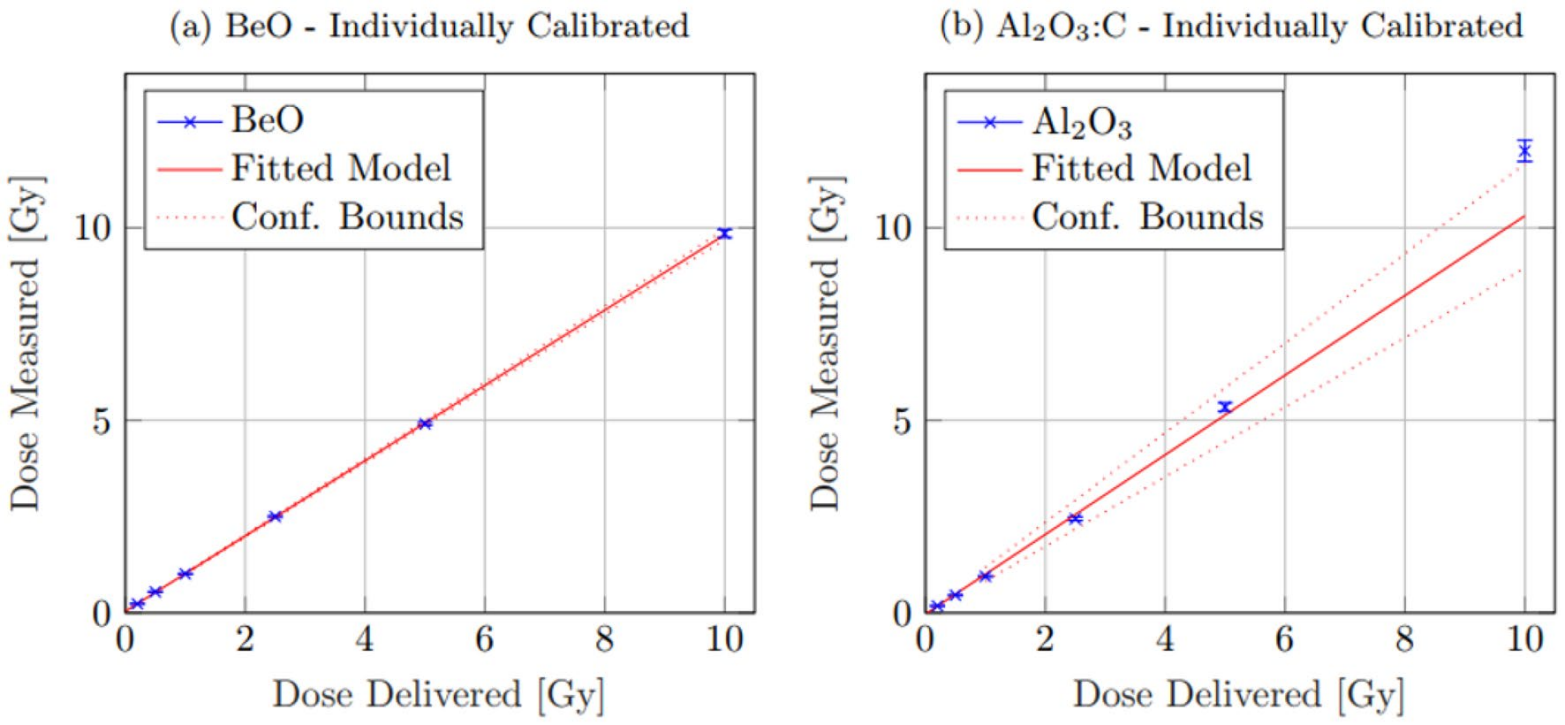
This figure shows the results of linear model fitting for both Al_2_O_3_:C and BeO when calibrated against the individual OSLD response (**a**, **b**). While a slight increase in uncertainty is apparent in the data from Al_2_O_3_:C when individually calibrated, there is a substantial decrease in that of the BeO, resulting in a much tighter fit

Further, while it seems Al_2_O_3_:C has a more precise value directly from group mean normalisation, after 5 Gy, there appeared to be a substantial deviation from linear behaviour. At 10.0 Gy, Al_2_O_3_:C exhibited a dose of $12.0\pm 0.2$ Gy, an over-response of $\sim$ 20%. Individual normalisation increased this uncertainty to $12.0\pm 0.3$ Gy. For BeO, when normalised to the group mean, a much more linear trend was apparent. However, the uncertainty in dose was considerably larger, increasing with dose, such that at 10.0 Gy, the observed measured result was $9.5\pm 0.7$ Gy. Individual normalisation, however, offset the individual sensitivity magnitude variance, finding a result of $9.9\pm 0.1$ Gy (Fig. 6).

Linear model fitting was performed on each dataset using weighted, least squares regression. Group mean calibration was performed for comparison, while uncertainty for BeO was higher than Al_2_O_3_:C, there was very little deviation from linearity within the measured dose range. As a result, the expectation dose was calculated as ${\text{Dose}}_{\text{BeO},\mathrm{ group}}=\left(0.97\pm 0.03\right)x+(0.4\pm 0.1)$ Gy. For Al_2_O_3_:C, due to the witnessed deviation from linearity above 5 Gy, attempted linear model fitting resulted in a model of ${\text{Dose}}_{{\text{Al}}_{2}{\text{O}}_{3},\mathrm{ group}}=\left(1.0\pm 0.1\right)x-(0.4\pm 0.6)$ Gy, demonstrating an order of magnitude greater uncertainty. Exclusion of the $10.0$ Gy data point due to its deviation from linearity, however, greatly increased the accuracy of the Al_2_O_3_:C model fitting with an adjusted model of ${\text{Dose}}_{{\text{Al}}_{2}{\text{O}}_{3},\mathrm{ group}\left(\mathrm{adj}\right)}=(1.00\pm 0.09)x-(0.02\pm 0.04)$ Gy, showing very good linearity up to 5 Gy.

Individual OSLD calibration showed almost no difference within uncertainty bounds in the predicted model for Al_2_O_3_:C with a result of ${\text{Dose}}_{{\text{Al}}_{2}{\text{O}}_{3},\mathrm{ ind}}=(1.0\pm 0.1)x-(0.3\pm 0.5)$ Gy. The considerably smaller uncertainty bounds on each BeO data point, however, did result in a much tighter fit. A higher precision was achieved with a result of $Dos{e}_{BeO, ind}=(0.98\pm 0.01)x+(0.04\pm 0.01)$ Gy.

## Discussion

All readings were performed using the Lexsygsmart Automated TL/OSL reader. The reader can hold up to 40 OSLD or TLD and perform sequential read, bleach or annealing, at differing times, temperatures and stimulation light intensities, providing essentially unlimited permutations for reading and treatment cycles. For this reason, a pre-defined setting for reading and bleaching as described in the method was used for every case to maintain consistency across all measurements. Bleaching for 300 s was found to reduce the count rate to ~ 2000 counts/sec. Any longer than this appeared to have very little effect on residual signal. The carousel for housing the OSLD within the reader contains small, metal holding plates that are easily removed, such that they may be lifted from the carousel inside the machine for singular readout, ensuring individual treatment of each OSLD. It was noticed very early on that the position of the OSLD within the carousel affected the response. A difference in measured response of as much as $\sim$ 30% was observed from the same OSLD in a different carousel position. This was determined to be due to the differing reflectivity of individual holding plates, and so for all correlated measurements, it was ensured that the OSLD were placed in the same position using the same holding plate.

In the initial sensitivity comparison tests, while the response variance in individual Al_2_O_3_:C was generally quite small, with the largest difference between individual responses being $\sim$ 26%, BeO showed a much larger difference in the individual response of each OSLD of as high as $\sim$ 200%. Further, while the change in signal response over the first 16 exposures from new in Al_2_O_3_:C was $\sim$ 1%, the BeO showed an averaged decline in response of $\sim$ 45%. This initial decline in signal response and variance between individual OSLD response is demonstrated in Fig. 1. All BeO used were from the same production batch, and this sensitivity variance appears to occur even within the same batch. After 16 dose-bleach cycles, a generally steady BeO signal was observed of $\left(1.5\pm 0.9\right)\mathrm{ x }{10}^{5}$ counts. One of the key findings of interest is the improved precision when the individual OSLD were normalised to their own signal, rather than a group mean. For the case of BeO, this substantially improved uncertainty by minimising variance between OSLD response within the group. This same method, however, resulted in a general uncertainty seen from Al_2_O_3_:C. As seen in Fig. 3, BeO shows an apparent exponential decline of $Respons{e}_{BeO}=(0.55\pm 0.05){e}^{-\left(0.40\pm 0.05\right)x}+(0.54\pm 0.01)$ with little deviation, becoming much more stable after $\sim$ 12 cycles. However, the individual variance in Al_2_O_3_:C relative to initial response is substantially larger and less predictable, resulting in a much larger uncertainty when considering group averages after singular calibration. While BeO showed no significant difference in signal based on orientation for reading, there is reported information that for nanoDots placed in the Landauer MicroStar™ reader in the wrong orientation may result in an error of up to 11% [4]. A study of this potential orientation effect performed by irradiating five nanoDots at 1.0 Gy and 2.0 Gy, followed by flipping the OSLD between subsequent reads, showed no significant difference and so is not believed to have contributed to individual response variance.. Fading from multiple reads was found to be very minor for both OSLD, $<3$% after ten read cycles.

To test the linearity of dose–response, each OSLD received three exposures at each 0.2 Gy, 0.5 Gy, 1.0 Gy, 2.5 Gy, 5.0 Gy and 10.0 Gy. The maximal dose was chosen to be 10.0 Gy as this was designed as a preliminary study. This dose range encompasses most of the regular fractional dose deliveries in a clinical setting. Higher doses may be considered as part of a future study. The response was calibrated first to the group mean value at the initial 1.0 Gy delivery, and second, singularly calibrated to the initial individual OSLD response at 1.0 Gy. The measured dose was then taken as an average of all exposures of each OSLD type at each energy. As expected, due to sensitivity variance within the group, when normalised to the group mean, BeO showed large uncertainty, increasing with dose, such that at 10.0 Gy, the recorded dose was found to be $9.5\pm 0.7$ Gy, an uncertainty of $\sim$ 7.4%. In the same regime, the Al_2_O_3_:C demonstrated a much smaller uncertainty; however, while BeO appeared to remain linear in response over the range of measured doses, Al_2_O_3_:C began to show slight deviation from linearity after 2.5 Gy increasing after $5.0$ Gy, such that while a 10.0 Gy dose had an uncertainty of $\sim$ 1.6%, the measured dose value was $12.0\pm 0.2$ Gy and an over-response of $\sim$ 20%.

When individually calibrated, both OSLD appeared to follow the same trend of linearity; however, as with the sensitivity decline, the BeO showed a substantial decrease in uncertainty and increased accuracy. At the maximum 10.0 Gy dose, BeO recorded a measured dose of $9.9\pm 0.1$ Gy where the Al_2_O_3_:C OSLD appeared to have the same measured dose, however a slight increase in uncertainty to $12.0\pm 0.3$ Gy. BeO showed good linearity both group and individual calibration for the measured dose range with model fits of ${\text{Dose}}_{\text{BeO},\mathrm{ group}}=\left(0.97\pm 0.03\right)x+(0.4\pm 0.1)$ Gy and $Dos{e}_{BeO, ind}=(0.98\pm 0.01)x+(0.04\pm 0.01)$ Gy. Linear model fitting for Al_2_O_3_:C across the full 10 Gy measure range, however, showed much larger confidence bounds with models of ${\text{Dose}}_{{\text{Al}}_{2}{\text{O}}_{3},\mathrm{ group}}=\left(1.0\pm 0.1\right)x-(0.4\pm 0.6)$ Gy and ${\text{Dose}}_{{\text{Al}}_{2}{\text{O}}_{3},\mathrm{ ind}}=(1.0\pm 0.1)x-(0.3\pm 0.5)$ Gy for each group and individual calibration, respectively. Exclusion of the 10 Gy data point for Al_2_O_3_:C did however result in a much tighter bound model up to 5.0 Gy of ${\text{Dose}}_{{\text{Al}}_{2}{\text{O}}_{3},\mathrm{ group}\left(\mathrm{adj}\right)}=(1.00\pm 0.09)x-(0.02\pm 0.04)$ Gy.

While variation between individual BeO dosimeters was deemed to be of little concern due to being systematic and able to be compensated for with individual calibration, future studies would benefit from utilising a higher number of dosimeters to tighten bounds on random uncertainties, such as the variance seen after relative signal stability is achieved from initial use. Time constraints around this study made this difficult. Moving forward, future work will involve investigations into energy dependence of dose-response, signal fading with repeated use, signal response for lower kV X-ray energies and the possibility of `resetting' sensitivity after time through the TT-OSL phoenomena will aim to provide the basis for future, focused studies.

## Conclusion

In all, this has demonstrated that while directly from initial use with raw data, Al_2_O_3_:C shows an easier and more immediate use, an initial conditioning treatment to move into the approximately constant region of sensitivity decline in BeO, combined with singular calibration, show potentially higher accuracy and precision in measurements than Al_2_O_3_:C, making it a viable alternative. After this initial decline region, BeO showed a more stable and predictable response compared to Al_2_O_3_:C. While both OSLD types show good linearity up to $\sim$ 5 Gy, at this point, there is an apparent deviation from linearity to an over-response observed in Al_2_O_3_:C, where BeO maintains a strong linear relationship up to 10 Gy, conforming with literature and potentially providing advantages in clinical scenarios where higher absorbed dose may be expected. It is recommended in the case of both Al_2_O_3_:C and BeO that immediately prior to any clinical use, a calibration dosage is administered to maintain high accuracy and precision of response.

Future studies will aim to investigate energy dependence of dose-response, signal fading with repeated use, dose rate response and angular dependence, the possibility of measuring accumulated dose, and the link between BeO sensitivity variance and the recently discovered thermally transferred optically stimulated luminescence (TT-OSL) phenomena [12]. A similar study method as used here at energy ranges of 50–150 kV could be of potential benefit in this case. Finally, while at this stage, in-vivo dosimetry using TLD/OSLD is primarily used in first or early treatment deliveries as confirmation of calculated distributions, investigation of the TT-OSL response of BeO could have an advantage for monitoring an accumulated dose history for more than one exposure, or for recovery of OSL signal after bleaching should the need occur.
